# γδ T Cells Participating in Nervous Systems: A Story of Jekyll and Hyde

**DOI:** 10.3389/fimmu.2021.656097

**Published:** 2021-03-31

**Authors:** Yunxuan Li, Yixi Zhang, Xun Zeng

**Affiliations:** State Key Laboratory for Diagnosis and Treatment of Infectious Diseases, National Clinical Research Center for Infectious Diseases, Collaborative Innovation Center for Diagnosis and Treatment of Infectious Diseases, The First Affiliated Hospital, School of Medicine, Zhejiang University, Hangzhou, China

**Keywords:** γδ T cells, neuroinflammation, nociceptors, short-term memory, anxiety, IL-17

## Abstract

γδ T cells are distributed in various lymphoid and nonlymphoid tissues, and act as early responders in many conditions. Previous studies have proven their significant roles in infection, cancer, autoimmune diseases and tissue maintenance. Recently, accumulating researches have highlighted the crosstalk between γδ T cells and nervous systems. In these reports, γδ T cells maintain some physiological functions of central nervous system by secreting interleukin (IL) 17, and neurons like nociceptors can in turn regulate the activity of γδ T cells. Moreover, γδ T cells are involved in neuroinflammation such as stroke and multiple sclerosis. This review illustrates the relationship between γδ T cells and nervous systems in physiological and pathological conditions.

## Introduction

γδ T cells are T lymphocytes that express T-cell receptor gamma chain and delta chain to constitute γδ T-cell receptors (TCRs). Like conventional αβ T cells and B cells, γδ T cells also utilize somatic V, D, J gene rearrangement to express various TCRs for antigen recognition. Different from major histocompatibility complex (MHC) restricted manner, γδ TCRs follow antibody-like recognition manner to bind diverse ligands such as small and large, peptidic and non-peptidic, and foreign and self-molecules (1–3). Though γδ T cells contribute a minor population in the blood and lymphoid tissue, they are abundant in barrier tissue and their frequency in the blood can expand dramatically during infection (2). Using different V regions of γδ TCR chains, different subsets of γδ T cells reside in meninges, skins, lungs, livers, peritoneal cavity, adipose tissue, uterus, tongue, gut, blood and secondary lymphoid organs, depending on the different waves of γδ T cell development before and after the birth (4–9). Depending on TCR signaling strength during development, γδ T cells differentiate into two main effector subsets based on the types of cytokines produced: interferon-gamma (IFN-γ) and interleukin17 (IL-17) (γδ T17 cells) (5, 10). γδ T cells can participate in an immediate immune response as their direct antigen recognition, wide distribution, diverse ligands of γδ TCRs and expression of innate receptors. Indeed, tons of evidences have indicated that γδ T cells play pivotal roles in infection, tumor, autoimmunity and immune surveillance (7, 11–13). During the immune responses, γδ T cells can be activated by TCRs and/or by innate signals [e.g. cytokines and natural-killer group 2, member D (NKG2D)] (14). The clear roles of TCR signals and innate signals in regulating the functions of γδ T cells are still under debate. Regardless, the activated γδ T cells can secrete IL-17 to recruit neutrophils to amplify the inflammatory signals, promote the maturation of dendritic cells to prime αβ T cells, eliminate infected cells by directly releasing IFN-γ, perforin, granzyme B, and granulysin after sensing antigen and antibody-dependent cell-mediated cytotoxicity, and present antigen to αβ T cells (11). In addition, γδ T cells can repair damaged tissues by producing cytokines and chemokines, which is crucial for the homeostatic maintenance in the epidermis, intestinal epithelium, and adipose tissues (7). These tissue-resident γδ T cells show more functions than any other immune cells, as they can secrete insulin-like growth factor 1 (IGF-1) to improve epithelial cell survival and produce IL-17F to promote lipolysis and thermogenesis in adipose tissue (15, 16).

γδ T cells are also important for the inflammatory responses in neurological diseases. More importantly, new evidences have indicated that meningeal γδ T cells play important roles in maintaining the homeostasis of nervous system. These findings have suggested the complex role of γδ T cells in neuron-immune interactions. To better understand the functions of γδ T cells in nervous system, it is necessary to summarize the latest progress in the interaction between γδ T cells and nervous system. In this review, we will discuss how γδ T cells interact with nervous system in physiological and pathological conditions.

## γδ T Cells Interact With the Central Nervous System

**Figure 1:**
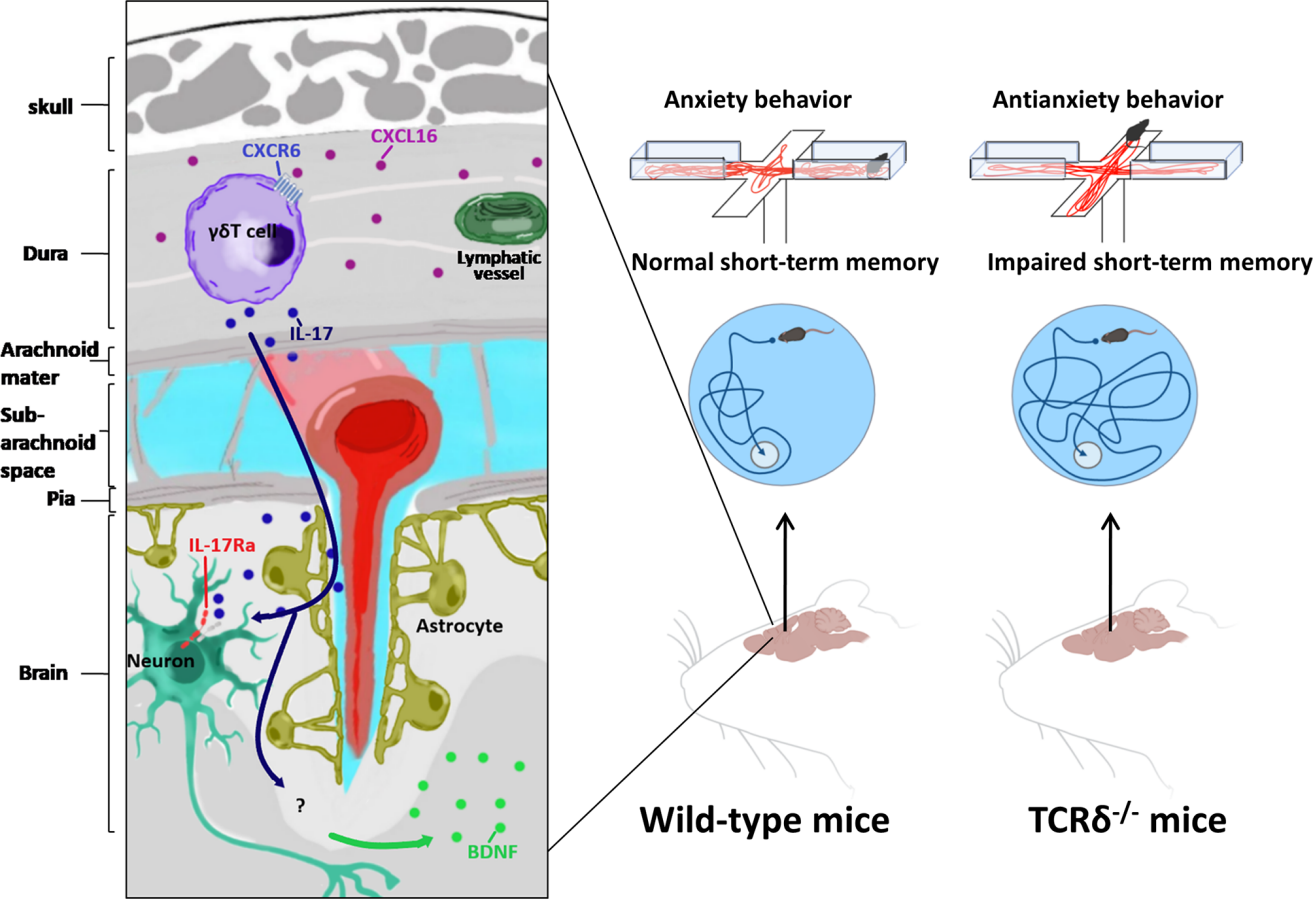
Meningeal γδ T cells involved in behavior regulation. Relying on CXCR6-CXCL16 axis, meningeal γδ T cells migrate to dura mater shortly after birth. Meningeal γδ T cells influence brain by constant secretion of IL-17. Neurons in medial prefrontal cortex(mPFC) and somatosensory cortex (S1DZ) express IL-17Ra in response to IL-17 to regulate anxiety-like behavior of mice. IL-17 induced glial brain-derived neurotrophic factor (BDNF) modulates hippocampal neuronal plasticity to maintain short-term memory.

For past decades, it has been conventionally believed that the central nervous system (CNS) has immune-privileged properties as it is shielded by the blood-brain barrier (BBB) that features with low expression of leukocyte adhesion molecules and tight junctions between brain capillary endothelial cells (17, 18). However, accumulating evidences have suggested that CNS and immune system can directly crosstalk with each other (19). Some immune molecules, such as cytokines, play a role in learning, memory and social behavior (20, 21). Moreover, a triple-layered membrane surrounding brain parenchyma called meninges is confirmed to bypass the BBB, which may be a place for immune surveillance and maintaining homeostasis of CNS (19). Along this line, diverse meningeal immune cells have been described in many articles. Among these immune cells, meningeal T cells were identified to secrete interleukin 4 (IL-4), interleukin 13 (IL-13), and IFN-γ, which correlate with learning, long-term memory and social behavior (20–22). IL-17, a key cytokine for inflammation, has also been discovered to administrate the fetal brain development and behavioral abnormalities (23, 24). Since meningeal γδ T cells have been identified as well, it is interesting to know the role of γδ T cells in regulating brain functions. Recently, two elegant works by Ribot’s group and Kipnis’ group found that meningeal γδ T cells could secrete IL-17 to regulate short-term memory and anxiety-like behavior, which partially addressed this issue (8, 25) (**Figure 1**).

Meningeal γδ T cells are tissue-resident cells expressing C-X-C Motif Chemokine Receptor 6 (CXCR6) and are attracted by chemokine C-X-C Motif Chemokine Ligand 16 (CXCL16), which is highly expressed in dura-resident myeloid cells (25). They migrate to meninges shortly after birth and are prevalent in dura mater. More importantly, they are the major source of IL-17 (8, 25). The majority of these meningeal γδ T17 cells are fetal derived Vγ6Vδ1 (the V region of TCR γ chain uses TRGV6 gene and the δ chain uses TRDV1 gene) T cells with canonical identical Vγ6-Jγ1 and Vδ1-Dδ2-Jδ2 chains that can be found in various non-lymphoid tissues (8, 9, 26, 27). Mice deficient γδ T cells and IL-17 showed impairments in short-term memory in tests of Y-maze and the Morris water maze. γδ T cell derived IL-17 can modulate the expression of neurotrophic factor (BDNF) in the hippocampus, which is able to regulate synaptic plasticity of neurons required for short-term memory (8). On the other hand, compared to WT mice, TCRδ^-/-^ mice and WT mice with the presence of anti-TCRδ antibodies in the Cerebrospinal fluid (CSF) showed severe anxiety-like behavior in the elevated plus maze and the open field (25). Collectively, these data suggested that meningeal γδ T17 cells played a key role in short-term memory and anxiety-like behavior. In this scenario, it is important to figure out the target of IL-17. Since IL-17 receptor A (IL-17RA) is expressed not only on astrocytes and microglial cells, but also on neurons throughout all cortical layers of the medial prefrontal cortex (mPFC) and somatosensory cortex (S1DZ), the observation that conditional knockout of IL-17RA on astrocytes and microglial cells did not disturb short-term memory suggested that IL-17 could directly affect neurons (8, 25, 28). Indeed, IL-17 signaling affected mPFC neurons by down-regulating the activity of gamma-aminobutyric acid (GABA)-benzodiazepine, the prototypical pathway for anxiolytic drugs (25). The further detailed molecular mechanism of how IL-17 signaling regulates short-term memory and anxiety-like behavior requires further study. Furthermore, unlike lungs, skins and guts, meninges do not have pathogenic or inflammatory stimuli in steady state (18, 29). How could meningeal γδ T cells continuously produce IL-17? It is found that the IL-17 production of meningeal γδ T cells is irrelative with pro-inflammatory cytokines interleukin 1 (IL-1) and interleukin 23 (IL-23), and pathogen-associated molecular pattern signals (8). In addition, it is still inconclusive whether components of commensal microbiota regulate IL-17 production of meningeal γδ T cells (8, 25). Therefore, the detailed mechanisms of continuous production of IL-17 by meningeal γδ T cells are still unclear.

## γδ T Cells Interact With the Peripheral Nervous System

As one of the most essential protective mechanisms of human body, nociceptive pain responds to chemical, mechanical, and thermal stimuli and can be detected by nociceptors around the body in peripheral nervous system (PNS) (30). As a particular subset of primary sensory neurons, nociceptors can respond to pain stimuli and subsequently convert the stimuli into nerve impulses to inform brain to produce the sensation of pain (31). Once receiving the stimuli, nociceptors can regulate the immune cell response activity at the tissue by releasing neuropeptides which were stored at the dense-core vesicles both in nociceptors’ synaptic terminals at the CNS and in the nerve endings within the peripheral tissues (32). The transducers of noxious stimuli are voltage-gated and ligand-gated ion channels expressed on the nociceptor nerve terminals, such as transient receptor potential vanilloid subfamily member 1 (TRPV1), transient receptor potential ankyrin 1 (TRPA1), Nav (Voltage-gated sodium channels)1.7, Nav1.8, and Nav1.9 (33).

**Figure 2:**
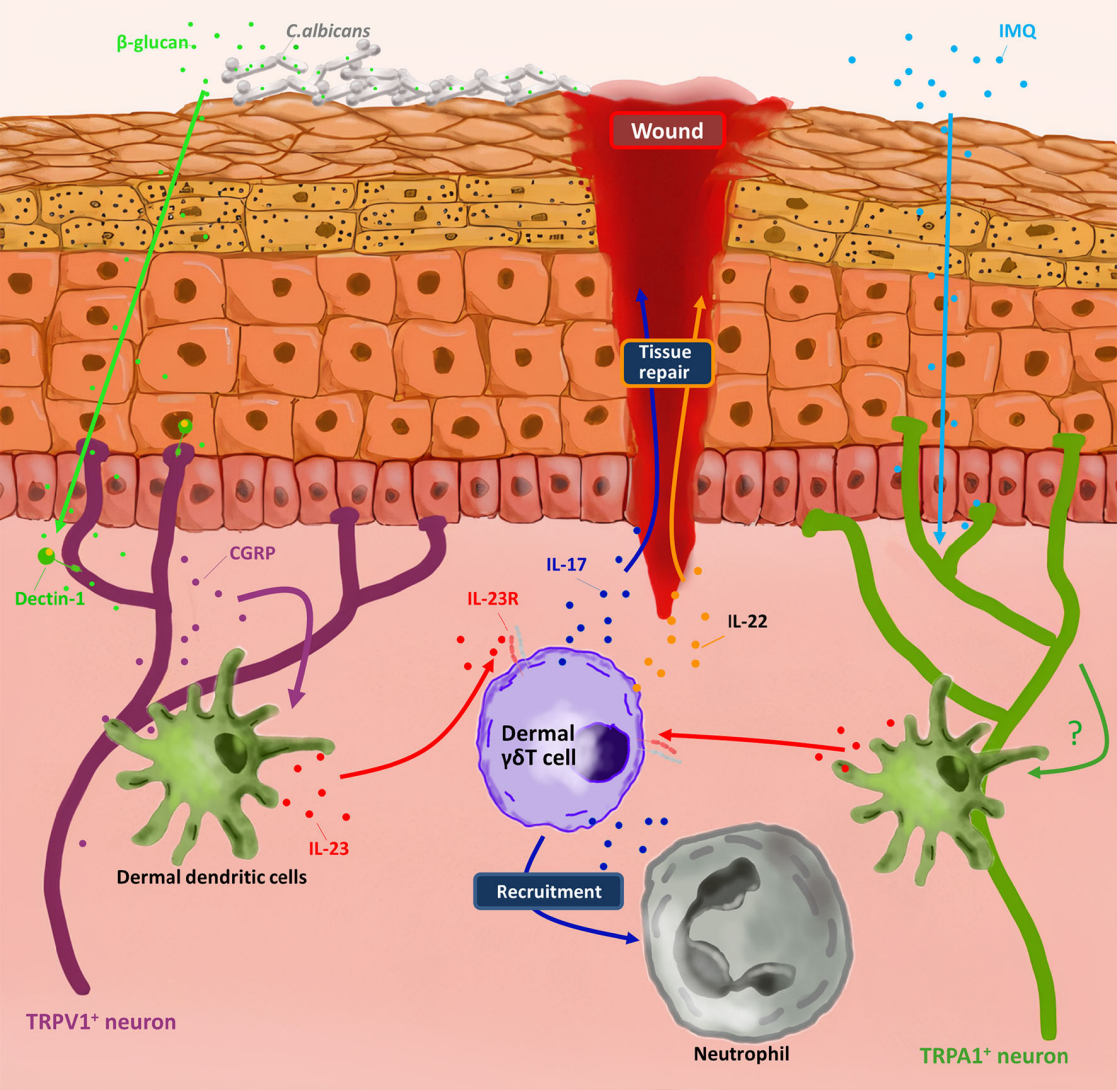
Dermal γδ T cells involved in nociceptors-induced skin protection. By sensing molecules such as *C. albicans*-derived soluble β-glucan and imiquimod (IMQ) respectively, TRPV1+ neuron and TRPA1+ neuron active dermal dendritic cells produce IL-23. In response to IL-23, dermal γδ T cells produce IL-17 and IL-22 to repair the wound and recruit neutrophils for pathogen clearance.

Among various immune cells, γδ T cells are also regulated b nociceptors. It has been reported that TRPV1^+^ and Nav1.8^+^ nociceptors were necessary factors to drive imiquimod (IMQ) induced psoriasis-like inflammation in skin by promoting dermal dendritic cells (dDCs) to produce Interleukin 23 (IL-23). IL-23-producing dDCs could activate IL-23 receptor positive (IL-23R^+^) dermal γδ T cells to secrete IL-17A, IL-17F and IL-22, which resulted in the recruitment of neutrophils to skin and hyperproliferation of keratinocytes (34, 35). Extended studies provided more details of how TRPV1^+^ nociceptors, dDCs and dermal γδ T cells interacted with each other in a fungus infection mouse model. TRPV1^+^ neurons were activated through Dectin-1 by sensing the β-glucan of *Candida albicans*, a kind of sugars on cell walls of fungus. Activated TRPV1^+^ neurons released neuropeptide calcitonin gene–related peptide (CGRP) to drive dDCs to produce IL-23, which could promote dermal γδ T cells to produce IL-17 and subsequently active downstream pathways to inhibit *C. albicans* infection (36–38). Ablating TRPV1^+^ nociceptors by resiniferatoxin (RTX) could reduce the numbers of IL-17 producing γδ T cells and the efficiency of *C. albicans* elimination (36). This finding, together with other findings that α-hemolysin of *Staphylococcus aureus* (*S. aureus*) and streptolysin S of *Streptococcus pyogenes* (*S. pyogenes*) can activate TRPV1^+^ nociceptors to secrete CGRP, indicated that some pathogen related molecules were sufficient for TRPV1^+^nociceptors activation and CGRP secretion as a consequence (39, 40). Therefore, Kaplan and colleagues tried to figure out whether the activation of TRPV1^+^ neurons alone could trigger γδ T17 response in a pathogenic molecule-free condition. By using optogenetic mouse model, they found that activation of TRPV1^+^ neurons alone sufficiently induced IL-17 production by γδ T cells in skin via releasing CGRP. More importantly, activated TRPV1^+^ neurons provided signals through nerve reflex arc that could induce γδ T17 response at adjacent, unstimulated skin (37). Therefore, neurons secreted molecules are perfectly capable of activating skin innate immune response, not only at the stimulated skin, but also at adjacent unstimulated skin, which may be benefit for limiting the infection. In addition, γδ T cells can cooperate with TRPA1^+^ nociceptors to promote systemic skin regeneration. Leung’s group found that in IMQ induced inflammation mouse model, TRPA1^+^ neurons, but not NLR family pyrin domain containing 3 (NLRP3), Toll like receptor 7 (TLR7) and TRPV1^+^ neurons, stimulated local IL-23 production by dDCs, thereby activating γδ T17 for tissue regeneration. Although the details of how TRPA1^+^ neurons promote dDCs to secrete IL-23 still need to be clarified, the results in mice with genetically defective of TRPA1^+^ neurons, γδ T cells and chemical removing dDCs suggested that none of these cells were redundant in skin wound healing (41). However, since both TRPA^+^ and TRPV^+^ neurons can activate γδ T17 cells via IL-23 secreted by dDCs, the reason why only TRPA^+^ neurons can promote γδ T17 cells for wound healing needs to be clarified. Other potential mechanisms may exist in wound healing by TRPA^+^ neurons regulated γδ T17 cells and are required for further investigation. Taken together, the axis of nociceptors-dDCs-γδ T17 cells has been discovered and plays a key role in defense against pathogen invasion and skin wound healing (**Figure 2**).

The crosstalk of nociceptors and γδ T cells is not always to protect the host against infection. In lethal *S. aureus* pneumonia mouse model, Chiu’s group found that TRPV1^+^neurons downregulated lung γδ T cells, resulting in a decrease in the recruitment of neutrophils that are essential for bacterial clearance. Mice ablating TRPV1^+^ nociceptors by RTX showed better survival rate and could increase lung γδ T cell number, in which the major increased γδ T cell subsets were Vγ1^+^ and Vγ1^-^Vγ2^-^ subtypes (42). Although the blockade of CGRP antagonist and the ablation of TPRV1^+^nociceptors have the similar phenotypes in the regulation of *S. aureus* pneumonia, the direct evidence of how CGRP regulates γδ T cells is still missing. Moreover, the specific role of IL-17 in this model has not been well described, even though the dynamic changes of neutrophils in TPRV1^+^nociceptors ablated mice have indicated that IL-17 played a pivotal role during the infection. In addition, Ghasemlou’s group tried to decipher the function of γδ T cells in different pain mouse models. Compared with WT mice, TCRδ^-/-^ mice had no differences in baseline sensitivity and mechanical or thermal hypersensitivity after injury, but with higher numbers of myeloid cells and monocytes. This finding suggested that γδ T cells did not contribute to the sensitization of inflammatory pain, but were involved in regulating the recruitment of myeloid cells and monocytes (43). Regardless, these data suggested that the mechanism of nociceptors regulating immune cells may be very complicated in different organs. A comprehensive study of how nociceptors regulate the whole immune system is required.

## γδ T Cells in Neurological Diseases

Neuroinflammation happens in the nervous system especially in the CNS, and is associated with most neurodegenerative disease (such as Parkinson’s disease (PD), multiple sclerosis (MS) and amyotrophic lateral sclerosis) (44–46). Several factors, such as autoimmunity, infection, injury and aging, may induce the incidence of neuroinflammation (47–49). At the beginning of neuroinflammation in the damaged tissue, recruited immune cells help to reconstruct tissue and repair neurons (50, 51). The persistent neuroinflammation results in chronic inflammation and neuronal death (52). As kick-starters of inflammation, γδ T cells participate in many neuroinflammation related diseases (53) (**Table 1**).

**Table 1:** The role of γδ T cells in neurological diseases.

Diseases	species	Role of γδ T cells	γδ T cell subsets	Main cytokines of γδ T cells	Related protein	References
MS	Human	Detrimental	Vδ1 /Vδ2 T cells	IFN-γ IL-17	• Migration: CXCR3, IP-10, CCL21.	(54–71)
EAE	Mouse	Detrimental	Vγ4/ Vγ6 T cells (natural and induced γδ T17 cells)	IL-17 IL-21 IL-15	• Migration: CD11a-d, CCR6, CCR2. • Stimulator: IL-23, IL-1β.	(72–93)
		Protective	Vγ1 T cells	IFN-γ CCL4	–	(93, 94)
Stroke	Human	Detrimental	–	IL-17	–	(95–97)
	Mouse	Detrimental	CCR6^+^γδ T cells (mainly Vγ6 T cells)	IL-17	• Synergy: TNF-α • Inhibition: IL-10 • Migration: CCL20, CCR6 • Stimulator: IL-23	(95–100)
IH	Human	Detrimental	–	IL-17	• Stimulator: hemoglobin, TLR2/TLR4 heterodimer, IL-23	(101)
RE	Human	Detrimental	Vδ1 T cells	TNF IFN-γ	–	(102, 103)
CM	Human	Protective	Vγ9Vδ2 T cells	Granzyme B	• Stimulator: granulysin. • Antigen: soluble phosphoantigens.	(104–106)
	Mouse	Protective	Vδ6.3 T cells	IFN-γ M-CSF CCL5 CCL3	–	(107–110)
		Detrimental	–	IFN-γ	–	(111)
WNV infection	Mouse	Protective	Vγ1 T cells	IFN-γ	–	(112, 113)
		Detrimental	Vγ4 T cells	IL-17 TNF-α	–	(114)

MS, multiple sclerosis; IFN-γ, interferon-γ; IL, interleukin; CXCR, CXC type chemokine receptor; IP-10, IFN-γ-induced protein 10; CCL, C-C motif chemokine ligand; EAE, experimental autoimmune encephalomyelitis; CCR, C-C chemokine receptor; CD, cluster of differentiation; GM-CSF, granulocyte-macrophage colony-stimulating factor; TNF, tumor necrosis factor; Th17, T helper 17; CCL, C-C motif chemokine ligand; DC, dendritic cells; TLR, Toll-like receptors; IH, intracerebral hemorrhage; RE, Rasmussen’s encephalitis; CM, cerebral malaria; WNV, West Nile Virus.

### Multiple Sclerosis and Experimental Autoimmune Encephalomyelitis (EAE)

As a potentially disabling disease of the CNS, multiple sclerosis (MS) is caused by the immune system attacking on protective myelin sheaths that cover neurons, eventually disabling the communication between brain and the rest of the body (45). Though many comparisons among patients with primary progressive MS, patients with relapsing-remitting MS (RRMS), healthy controls and patients with other neurological diseases have been done and the differences of the frequency of γδ T cells in the peripheral blood in patients with MS remains contradictory (54–58).While the percentage of Vδ2 T cells decreased and the percentage of Vδ1 T cells increased in peripheral blood of MS patients (58, 59), γδ T cells in MS patients expressed higher level of C-X-C Motif Chemokine Receptor 3 (CXCR3) that was related to the migration of T cells to MS plaques (60). As one of the two ligands of CXCR3, IFN-γ-induced protein 10 (IP-10) was elevated in both primary progressive MS and RRMS (61). Another ligand Chemokine (C-C motif) ligand 21 (CCL21) was remarkably reduced in the CSF during remission (62). For a better understanding of infiltrated γδ T cells in CNS, TCR repertoire analyzes were performed and the data revealed an oligo clonal expansion of γδ T cells in CNS of MS patients, suggesting these γδ T cells responded to common antigens (63–65). Along this line, the non-classical major histocompatibility complex (MHC) molecule CD1d, which could express lipid antigens to T cells, was found to be able to present myelin-derived glycosphingolipid antigen sulfatide in MS and recognized by γδ TCRs in sulfatide-specific manner (66). CD1d immunoreactivity was increased in MS, which suggested that one of early events in active phases of demyelination might be the lipid antigen presentation to γδ T cells (67). Furthermore, another potential γδ TCRs ligand, heat-shock protein 65 (HSP65), could induce γδ T cells expansion (68). Vδ1T cells could co-localize with HSP65^+^ oligodendrocytes within the sites of remyelination in MS lesions (69, 70). Studies further revealed that Vδ1 T cells expressed high level of IFN-γ that correlated with inflammation and nerve damage in newly diagnosed MS, whereas CD161^high^ CCR6^+^ γδ T cells (a γδ T cell subset expressing IL-17 in human) were enriched and produced IL-17 in the CSF of patients during relapse (58, 71). The component and function of γδ T cells have been extensively studied in EAE mouse model.

Experimental autoimmune encephalomyelitis (EAE) is one of widely used MS animal models that shares the same pathological feature including inflammation, demyelination, axonal loss and gliosis (115). Several immune response–modifying therapies have been successfully translated from EAE studies to clinical practice for MS treatment (116). IL-17 is indicated as a key pro-inflammatory cytokine in EAE, which is secreted by T helper 17 (Th17) cells and γδ T17 cells (72–74).The two cell types cannot be replaced by one another, as a reduced EAE severity was observed in either Th17 depletion or γδ T cell deficient mice (74, 75). In the process of EAE, γδ T cells expressed CD11a-d that might be essential for γδ T cell trafficking to the CNS, as indicated by the fact that deletion three out of four CD11 molecules dramatically reduced the severity of EAE (76–78). Moreover, γδ T17 cells with a downregulation CCR6 and an upregulation C-C Motif Chemokine Receptor 2 (CCR2) promoted the migration of γδ T cells to CNS in EAE (79). Therefore, these molecules promoted the rapid infiltration of γδ T17 cells into CNS and enabled them to be involved in early inflammation in EAE. Notably, a dynamic γδ TCR repertoire analysis indicated that most of infiltrated γδ T cells at the early phase of EAE were Vγ4Vδ6 and Vγ6Vδ1 with a highly focused γδ TCR repertoire, which has been reported as natural γδ T17 cells (nγδ T17) (74, 80–82). This data was consistent with early studies that the majority of infiltrated γδ T cells in the brain and spinal cord expressed Vγ1, Vγ4 and Vγ6 at the onset of EAE, while the majority of Vγ transcripts could be detected at the later phase, suggesting that different γδ T subsets participate the process of EAE (80). In addition to thymic-derived nγδ T17, peripheral γδ T cells, especiallyVγ4^+^T cells, can be induced to differentiate and produce IL-17 upon IL-23 stimulation in EAE (83). Both Vγ4 and Vγ6 T cells could produce high expression levels of IL-1 receptor (IL-1R) and IL-23R to bind activated monocytes and dendritic cells secreted IL-1β and IL-23 to release IL-17 and interleukin 21 (IL-21), which could facilitate Th17 cells to produce IL-17, IL-22 and granulocyte-macrophage colony stimulating factor (GM-CSF) to exacerbate neuroinflammation (74, 83–85). IL-17 could also stimulate BBB endothelial cells, microglia and astrocytes to release multiple cytokines and chemokines to recruit neutrophils to breakdown BBB, and finally, to attract various leukocytes into the CNS (86–89). IL-23-activated γδ T cells could not only promote Th17 cells function, but also restrained the conversion of naïve T cells to Tregs and suppressed the Treg responses to enhance inflammation (74, 90). In addition, a subset of interleukin 15 (IL-15)-secreting γδ T cells was found to induce CD44^high^ memory T cells by releasing IL-15 and help to switch memory T cells to Th17 cells to induce EAE (91). However, not all γδ T cells were inflammatory signals promoter. IFN-γ producing γδ T cells, majority of which were Vγ1 T cells, induced IFN-γ expression by encephalitogenic T cells, suppressed the activity of Th17 and released Chemokine (C-C motif) ligand 4 (CCL4) to recruit C-C Motif Chemokine Receptor 5 (CCR5) ^+^ Tregs to reduce the inflammatory signals (92, 93). Additionally, γδ T cells regulated inflammation through Fas/Fas ligand, which could induce encephalitogenic T cells apoptosis and facilitate the recovery from EAE (94). Regardless, given that the infiltrated γδ T cells highly expressed IL-17 in the CNS and amplified Th17 responses, it was recognized that γδ T cells were more pathogenic than protective, especially in the early stage of the diseases (74). Therefore, Therapies targeting IL-17, IL-17 receptor (IL-17R) or upstream cytokines IL-1β or IL-23 would not only suppress Th17 and γδ T17 cells function, but also blocked the positive feedback loop between Th17 and γδ T17 cells (117–119). Indeed, several clinical trials targeting IL-17 have already shown encouraging results in relapsing remitting MS patients (117, 120, 121). As a potential therapeutic target, γδ T cells are required for more detailed investigation.

### Stroke

Stroke ranks second as the leading cause of death and third as the cause of disability all over the world. As a main kind of stroke, Ischemic stroke results from the middle cerebral artery occlusion, followed by brain tissue damage in the affected territory, which is caused by inflammatory response (122). Pathogenic mechanisms of γδ T cells in stroke are mainly due to the production of IL-17 (123). In human brain tissues, immunohistochemistry staining for γδ T cells and IL-17 showed the presence of γδ T cells and the production of IL-17 shortly after stroke (95). In addition, compared to healthy control, patients with stroke have increased level of IL-17 in peripheral blood (96). In rodent models, γδ T cells, rather than Th17 cells, was found as the major IL-17 producers in ischemia-reperfusion (I/R) injury (123). In this scenario, IL-23 is found to be a key cytokine to induce IL-17 production by γδ T cells during the delayed phase of ischemia. The mice with the deficiency of IL-23 or IL-17 had significantly reduced infarct size, whereas mice treated with IL-17 neutralizing antibodies within 3 hours of stroke had a better prognosis (95, 123). Further studies illustrated that interferon regulatory factor 4 (IRF4) ^+^/CD172a^+^ conventional type 2 DCs infiltrate into the ischemic brain rapidly and became the major source of IL-23 within 24 hours to stimulate CCR6^+^ γδ T cells (mainly Vγ6 T cells) to express IL-17 (97, 98). The absence of CD11c^+^ cells or the impaired IL-23 signaling could abrogate the production of IL-17 by γδ T cells (97). Genetic deficiency in *Ccr6* significantly diminished the infiltration of γδ T cells, highlighting the important role that chemokine (C-C motif) ligand 20 (CCL20)/CCR6 axis plays for γδ T cell migration in stroke (98). γδ T17 cells are not the only source of IL-17, astrocyte-derived IL-17 A facilitates survival and neuronal differentiation of neural precursor cells in the recovery phase of stroke (99). After synergistic stimulation of IL-17 produced by γδ T cells and TNF-α produced by macrophages, astrocytes secrete chemokines, such as CXCL-1, to facilitate the infiltration of neutrophils, thereby inducing matrix metalloproteinase 3 (MMP3) and MMP9, which were involved in the destruction of the BBB (95, 98). Blocking the signal of IL-17 or CXCL-1/CXCR2-axis could inhibit the invasion of neutrophils and improve neurological prognosis (95). It is worth noting that intestinal γδ T17 cells could migrate to the meninges to induce ischemic neuroinflammation by producing IL-17 after stroke. Intestinal dysbiosis affected stroke through γδ T cells by inhibiting intestinal γδ T17 cells trafficking from gut to meninges (100). After the treatment of antibiotics, the altered intestinal commensal bacteria activated CD103+ DCs in mesenteric lymph node, thereby inducing Tregs expansion and secreting the anti-inflammatory cytokine interleukin 10 (IL-10), which could suppress the differentiation of γδ T17 cells in lamina propria of the small intestine (100). Interestingly, as aforementioned, most of meningeal γδ T17 cells were Vγ6Vδ1T cells and the secretion of IL-17 contributed to the physiological functions of the brain (8). Moreover, commensal microbiota might conduce to IL-17 production of meningeal γδ T cells (25). Therefore, it is interesting to know whether meningeal γδ T17 cells are the main source of infiltrated γδ T cells into the ischemic brain and how commensal microbiota affect IL-17 production of meningeal γδ T cells directly or indirectly. Moreover, since IL-17 plays a key role in the progression of stroke, it can be a therapeutic target to reduce the severity of stroke (99). Controlling commensal microbiota may also benefit for the prognosis of stroke.

### Injury Related Neuroinflammation

The mechanical injury induced neuroinflammation in CNS is normally the outcome of BBB breakdown and inflammatory immune cells infiltration. For example, during intracerebral hemorrhage, hemoglobin from the hematoma can activate macrophages via Toll like receptor 2 (TLR2)/Toll like receptor 4(TLR4) heterodimer, which can secrete IL-23 to induce γδ T cells to produce IL-17 to aggravate secondary damage (124). Brain damage of Periventricular leukomalacia is also partially attributed to γδ T cells through the IL-17/IL-22 unrelated signaling pathways (101). While in Spinal cord injury, γδ T cells are sources of producing IFN-γ to aggravate lesions in the early phase (125). Moreover, traumatic brain injury has been linked with γδ T cells in the gut, for their increasing frequency after fluid percussion injury (126). Notably, most of mechanical injury induced neuroinflammation is pathogen free in CNS, suggesting that pattern recognition receptors (PRRs) expressed on γδ T cells are important signals for their activation. Therefore, further investigation is required to reveal how the respective and/or integrated TCRs and PRRs signals regulate γδ T cells function in CNS.

### Neurodegenerative Disease

PD is a chronic neurodegenerative disease that leads to a detrimental result of the CNS, especially the motor nervous system. The most important pathological features of PD are the degeneration of dopaminergic neurons in the substantia nigra and the accumulation of unique cytoplasmic inclusions (Lewis bodies) containing α-synuclein (44). A few preliminary correlations between PD and γδ T cells have been documented clinically. Compared to some other neurological diseases and tension headache, a higher proportion of γδ T cells was observed in the CSF in patients with PD (127). The frequencies and total numbers of γδ T cells were significantly decreased in the blood of PD group than that in healthy control group (128). In addition, γδ T cells partially expressed CD25 in the CSF of PD patients whereas they hardly expressed CD25 in blood, indicating a preferential activation of γδ T cells in the CSF (127). The relation between γδ T cells and PD might rely on microglia, which serve as tissue-resident macrophages within the brain. Stimulated through TLR2, TLR4, TLR7 or TLR9, microglia can release IL-1β and IL-23 to active γδ T cells to produce IL-17 *in vitro* (129). And neuron-released α-synuclein could directly bind TLR2 and trigger inflammatory responses in the microglia (130). TLR2 was additionally expressed on γδ T cells and exhibited co-stimulatory effects for activated γδ T cells (131). α-synuclein may be important for γδ T cells to participate in the PD. In addition, as another main neurodegenerative disease, Alzheimer’s disease (AD) is also connected with γδT cells. Clonotypes of TCR γ chain are more specific in patients with AD and in the brain compared with that in peripheral blood (132).

### Rasmussen’s Encephalitis

Rasmussen’s encephalitis (RE), especially occurring in children under the age of 10, is a rare chronic inflammatory neurological disease without the involvement of pathogenic microorganisms that feature with progress local atrophy of the cerebral cortex on unilateral cerebral hemisphere, refractory epilepsy and cognitive impairment (133). The majority of infiltrated T cells are cytotoxic CD8^+^ T cells and CD4^+^ T cells. γδ T cells can also be found in brain and they contribute to the secretion of TNF and IFN-γ. The ratio of γδ T cells to αβ T cells is obviously higher in brain-infiltrating lymphocytes than that in peripheral blood (134). The same TCRδ1 chain with the identical third complementarity determining region (CDR3) sequences was found in the brains of RE patients, suggesting that γδ T cells might respond to the same antigen(s) and be clonally expanded. What’s more, the same γδ TCR clones were found in focal cortical dysplasia (FCD), a disease with congenital abnormality of brain development, implying that the ligands recognized by γδ TCRs were more likely to come from self-antigens rather than foreign antigens (102). Identifying the potential γδ TCR ligands may be benefit for investigating the function of γδ T cells in RE or related diseases in CNS.

### Infection Related Neuroinflammation

As a lethal neurological complication of *Plasmodium* infection, cerebral malaria (CM) is responsible for the majority of child mortality (103). γδT cells can protect against *Plasmodium* infection by killing extracellular merozoites and intracellular late-stage parasites and regulating other lymphocytes such as αβ T cells and dendritic cells in both human and *Plasmodium* infection mouse model (104–109, 135). However, IFN‐γ producing γδ T cells in the liver stage of infection are responsible for experimental cerebral malaria (ECM). This proportion of liver γδ T cells promote a proinflammatory microenvironment to activate CD4^+^ and CD8^+^ T cells (110). These functional CD4^+^ and CD8^+^ T cells subsequently migrate to the brain and cause neuroinflammation resulting in ECM (110, 111). Besides, the parasites become more virulent in the presence of liver IFN‐γ producing γδ T cells to induce more pathogenic inflammation causing ECM development (110). In TCRδ^-/-^ mice or mice injected antibody to deplete γδ T cells, the CM development can be partially inhibited (110, 136).

West Nile Virus (WNV) infection is lethal for the induction of encephalitis (137). In WNV infected mice, γδ T cells play a dual role (138). On the one hand, γδ T cells can eliminate infected cells and contribute to the maturation of DCs to prime αβ T cells (112, 114). In this case, IFN-γ producing Vγ1^+^ T cells are able to limit the dissemination of WNV and prevent mortal WNV encephalitis (114, 138). On the other hand, γδ T17 cells (mainly Vγ4^+^T cells) can suppress the proliferation of Vγ1^+^ T cells, produce IL-17 and TNF-α to enhance BBB permeability and finally induce encephalitis (138). Similarly, γδ T17 cells have the same detrimental effects in the infection induced neuroinflammation in the mouse model of *Angiostrongylus cantonensis* infection. Among them, γδ T17 cells contributed to demyelination of the brain (113).

Perinatal infection can cause cerebral white matter injury in infants. In LPS-induced sepsis of postnatal days’ mice, it was γδ T cells, rather than αβ T cells, that contributed to white matter injury and subsequent abnormal motor function (139). Taken together, as one of the earliest immune responders, γδ T cells secret variety of cytokines to defend or exacerbate the infection in CNS.

## Conclusion

Most studies are focused on the detrimental or protective effects of γδ T cells in the diseases of nervous system. Here, we have also reviewed that the meningeal γδ T17 cells can support the short-term memory and anxiety-like behavior of mice, and the nociceptors induced activating or suppressing reactivity on dermal or lung γδ T cells. However, it is just a beginning. The reactions between γδ T cells and nervous system are far more than what these studies have reported. For example, meningeal γδ T cells will increase the expression of IL-17A to modulate anxiety-like behavior after the injection of LPS, indicating the possible link between meningeal γδ T cells and microbiota (25). It is interesting to know the role of meningeal γδ T cells play in gut-brain axis crosstalk. In addition, nociceptors regulate immune system (including γδ T cells) to respond infection (32). In this condition, the details of how neurons interact with immune cells are still missing. Therefore, further investigations about γδ T cells and behaviors, neuron-immune interactions in various disease models are required. More systematic researches need to be performed to reveal their relationships.

## AuthorContributions

YL and XZ drafted the main body of this manuscript. YZ modified the manuscript. XZ takes primary responsibility for this paper as the corresponding author. All authors contributed to the article and approved the submitted version.

## Funding

This work was supported by National Natural Science Foundation of China General Program (31870899).

## Conflict of Interest

The authors declare that the research was conducted in the absence of any commercial or financial relationships that could be construed as a potential conflict of interest.

## Abbreviation

TCR, T-cell receptors; MHC, major histocompatibility complex; IFN, interferon; IL, interleukin; IGF, insulin-like growth factor; CXCR, CXC type chemokine receptor; CXCL, C-X-C Motif Chemokine Ligand; CCL, C-C motif chemokine ligand; CCR, C-C chemokine receptor; IP-10, IFN-γ-induced protein 10; CD, cluster of differentiation; GM-CSF, granulocyte-macrophage colony-stimulating factor; TNF, tumor necrosis factor; BBB, blood-brain barrier; BDNF, neurotrophic factor; WT, wild type; mPFC, medial prefrontal cortex; S1DZ, somatosensory cortex; GABA, gamma-aminobutyric acid; PNS, peripheral nervous system; TRPV1, transient receptor potential vanilloid subfamily member 1; TRPA1, transient receptor potential ankyrin 1; NLRP3, NLR family pyrin domain containing 3; IRF, interferon regulatory factor; IMQ, imiquimod; DC, dendritic cells; RTX, resiniferatoxin; CGRP, calcitonin gene–related peptide; PD, Parkinson’s disease; MS, multiple sclerosis; RRMS, relapsing-remitting MS; EAE, experimental autoimmune encephalomyelitis; Th17, T helper 17; CSF, Cerebrospinal fluid; HSP, heat-shock protein; CNS, central nervous system; FCD, focal cortical dysplasia; CDR3, complementarity determining region; IH, intracerebral hemorrhage; CM, cerebral malaria; ECM, experimental cerebral malaria; RE, Rasmussen’s encephalitis; TLR, Toll-like receptors; PPR, pattern recognition receptor; MMP, matrix metalloproteinase; WNV, West Nile Virus; LPS, lipopolysaccharide.
